# The synthetic cobalt vanadium selenite, Co_2_V_2_Se_2_O_11_

**DOI:** 10.1107/S1600536812027286

**Published:** 2012-06-30

**Authors:** Faiz Rabbani, Iwan Zimmermann, Mats Johnsson

**Affiliations:** a Department of Materials and Environmental Chemistry, Stockholm University, SE-106 91 Stockholm, Sweden

## Abstract

The crystal structure of dicobalt(II) divanadium(V) disel­enium(IV) undeca­oxide, Co_2_V_2_Se_2_O_11_, exhibits a three-dimensional framework, the building units being distorted CoO_6_ octa­hedra and VO_5_ square pyramids arranged so as to form alternate chains along [010]. The framework has channels along [100] and [010] in which the two Ψ-SeO_3_ *E* (site symmetries *m*; *E* being the 4*s* ^2^ lone electron pair of Se^IV^) tetra­hedra reside and connect to the other building blocks. The structure contains corner- and edge-sharing CoO_6_ octa­hedra, corner- and edge-sharing VO_5_ square pyramids and edge-sharing Ψ-SeO_3_ *E* tetra­hedra. Co_2_V_2_Se_2_O_11_ is the first oxide containing all the cations Co^II^, V^V^ and Se^IV^.

## Related literature

For general background, including bond-valence-sum calculations, see: Brown & Altermatt (1985 ▶). For related structures, see: Allen *et al.* (2004 ▶); Becker *et al.* (2007**a* ▶,b* ▶); Jiang *et al.* (2008 ▶); Millet *et al.* (1999 ▶); Pitzschenke & Jansen (2007 ▶); Sauerbrei *et al.* (1974 ▶).

## Experimental

### 

#### Crystal data

Co_2_V_2_Se_2_O_11_
*M* *_r_* = 553.66
Monoclinic, 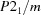

*a* = 4.7913 (2) Å
*b* = 8.8680 (4) Å
*c* = 10.6156 (5) Å
β = 101.115 (5)°
*V* = 442.59 (3) Å^3^
*Z* = 2
Mo *K*α radiation
μ = 14.01 mm^−1^
*T* = 292 K
0.05 × 0.03 × 0.02 mm

#### Data collection

Oxford Diffraction Xcalibur Sapphire3 diffractometer
Absorption correction: analytical [*CrysAlis RED* (Oxford Diffraction, 2007 ▶), based on expressions derived by Clark & Reid (1995 ▶)] *T*_min_ = 0.659, *T*_max_ = 0.756
4219 measured reflections
1534 independent reflections
1023 reflections with *I* > 2σ(*I*)
*R*_int_ = 0.051

#### Refinement

*R*[*F* ^2^ > 2σ(*F* ^2^)] = 0.032
*wR*(*F* ^2^) = 0.048
*S* = 0.76
1534 reflections
85 parameters
Δρ_max_ = 1.28 e Å^−3^
Δρ_min_ = −0.98 e Å^−3^

### 

Data collection: *CrysAlis CCD* (Oxford Diffraction, 2007 ▶); cell refinement: *CrysAlis RED* (Oxford Diffraction, 2007 ▶); data reduction: *CrysAlis RED*; program(s) used to solve structure: *SHELXS97* (Sheldrick, 2008 ▶); program(s) used to refine structure: *SHELXL97* (Sheldrick, 2008 ▶); molecular graphics: *DIAMOND* (Brandenburg, 2001 ▶); software used to prepare material for publication: *enCIFer* (Allen *et al.*, 2004 ▶).

## Supplementary Material

Crystal structure: contains datablock(s) I, global. DOI: 10.1107/S1600536812027286/pk2421sup1.cif

Structure factors: contains datablock(s) I. DOI: 10.1107/S1600536812027286/pk2421Isup2.hkl

Additional supplementary materials:  crystallographic information; 3D view; checkCIF report

## Figures and Tables

**Fig. 1.**
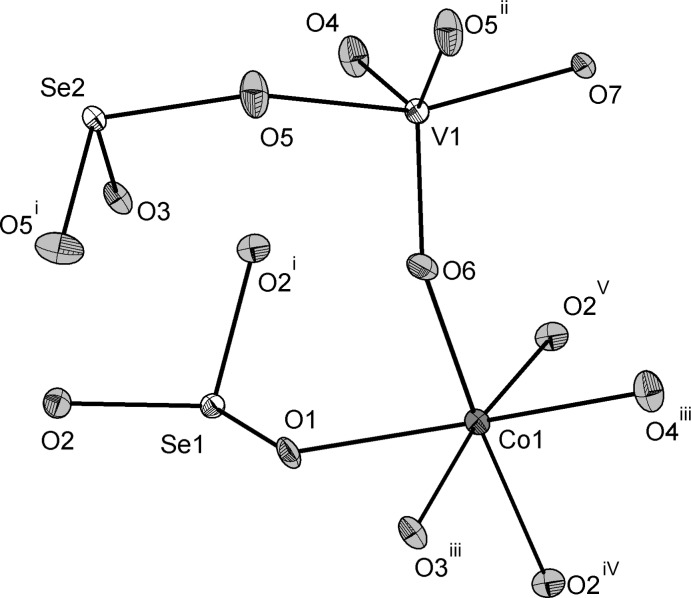
A displacement ellipsoid diagram showing the coordination around the cations. Atomic displacement parameters are given at the 50% probability level [Symmetry codes: (i) x,-y + 1/2,z; (ii) -x + 1,-y - 1,z - 2; (iii) x + 1,y,z; (iv) x + 1,-y + 1/2,z; (v) -x + 1,y + 1/2,-z + 1].

**Fig. 2.**
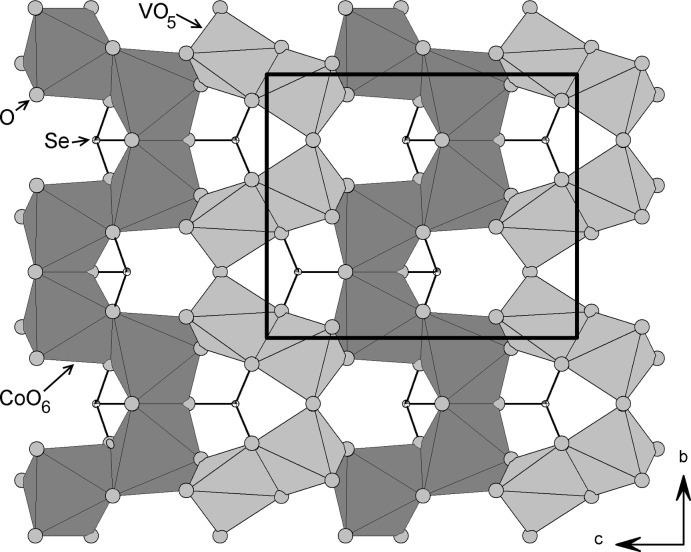
A view of the structure of Co2V2Se2O11 along (100).

**Fig. 3.**
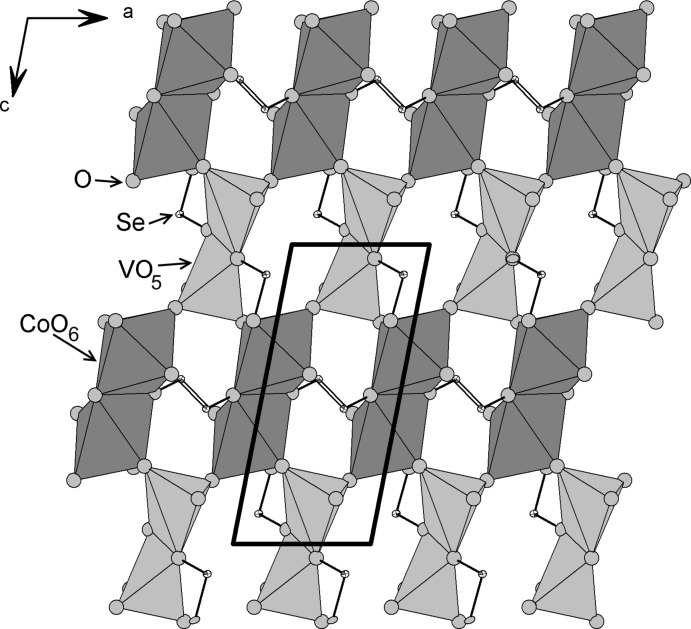
A view of the structure of Co2V2Se2O11 along (010).

## supplementary crystallographic information

### Comment

The synthesis and crystal structure determination of the new compound Co_2_V_2_Se_2_O_11_ is a result of an ongoing investigation of the structural chemistry of selenium and tellurium oxides and oxohalides. Transition metal oxides and oxohalides containing *p*-block cations, such as Se^IV^ and Te^IV^, with stereochemically active lone pairs frequently show a low-dimensional arrangement of the metal ions. The Se^IV^ lone pairs act as 'chemical scissors' and help to reduce the dimensionality of the atomic arrangements in the crystal structure (Becker *et al.*, 2007**a*,b*). The aim of the present study was to test the synthesis concept in a system containing two transition metals taking different coordination polyhedra: CoO-V_2_O_5_—SeO_2_. The present compound is, to the best of our knowledge, the first oxide to contain all the cations Co^II^, V^V^ and Se^IV^. A few selenites containing vanadium plus another transition metal have previously been described; Cd_6_V_2_Se_5_O_21_ (Jiang *et al.*, 2008), α-CuVSe_2_O_7_ and β-CuVSe_2_O_7_ (Millet *et al.*, 1999), AgVSeO_5_ and ZnVSe_2_O_7_ (Pitzschenke & Jansen, 2007). The stereochemically active lone electron pairs open up the crystal structures by creating non-bonding volumes and occupy space similar to that taken by an oxygen anion in the crystal structure. α-CuVSe_2_O_7_ is a layered compound with only weak van der Waals interactions in between the layers while the others are three-dimensional framework structures where the lone pairs on Se^IV^ are located in voids in the crystal structure.

Co_2_V_2_Se_2_O_11_ crystallizes in the centrosymmetric monoclinic space group P2_1_/m. The Co^II^ ions are in a slightly distorted octahedral environment. The CoO_6_ octahedra are connected *via* O2^iii^···O2^v^ and O1···O3^iii^ edge sharing [symmetry code: (iii) *x* + 1,*y*,*z*; (v) -*x* + 1,*y* + 1/2,-*z* + 1] to form zigzag chains having the formula [CoO_4_]_n_ along [100]. The Co···O bonds are in the range 2.074 (3) Å to 2.123 (3) Å which is comparable to what is found in *e.g.* Co_2_V_2_O_7_ (Sauerbrei *et al.*, 1974). There are two Co—Co distances within a chain; 3.160 (1) Å and 3.179 (2) Å. The V^V^ atoms are surrounded by five O atoms forming a very distorted square pyramid comprising two V1=O vanadyl double bonds of 1.627 (3) Å and 1.635 (3) Å and two long V1–O bonds 2.038 (3) Å and 2.087 (3) Å (Fig. 1). As a consequence, the V atom is located above the square plane. Bond valence sum analysis (Brown & Altermatt, 1985) confirms the coordination, with the calculated valence (V^V^, the bond valence parameter *R*_o_ = 1.803) equal to 5.15. The VO_5_ square pyramids are connected *via* O5···O5^ii^ edge sharing and O7 corner sharing [symmetry code: (ii) -*x* + 1,-*y* + 1,*z* - 2] to form chains having the formula [V_2_O_7_]_n_ along [010]. The V···V distances in a chain are 3.328 (1) Å and 3.443 (2) Å. The chains of CoO_6_ and VO_5_ bridge by corner sharing at O4 and O6. The Se^IV^ ions have both one sided SeO_3_ coordination owing to the presence of the 4s^2^ stereochemically active lone pair, E, and they do not polymerize. The crystal structure can be described as being a three-dimensional framework structure made up of [CoO_4_]_n_– and [V_2_O_7_]_n_ chains (Fig. 2 and 3). The SeO_3_ groups connect to the metal-oxide framework by corner sharing (Fig. 3). The stereochemically active Se^IV^ lone pairs are located in voids in the structure. Those voids are present as tunnels along (100) and (010) respectively.

Bond valence sum analysis (Brown & Altermatt, 1985) confirms the coordination for all the ions and give 2.04 for Co1, 5.15 for V1, 4.01 for Se1, 3.84 for Se2, 2.07 for O1, 2.02 for O2, 2.11 for O3, 1.92 for O4 and 2.36 for O5.

### Experimental

Single crystals of Co_2_V_2_Se_2_O_11_ are non-hygroscopic and were synthesized *via* chemical vapour transport reactions in sealed evacuated silica tubes. The starting materials were 0.150 g (2 mmol) CoO (ABCR GmbH 97.999%), 0.182 g (1 mmol) V_2_O_5_ (ABCR GmbH 99.9%), and 0.222 g (2 mmol) SeO_2_, (Alfa Aesar 99.4%) mixed in a stoichiometric 2:1:2 molar ratio and placed in a 5 cm long silica tube which was first dried for 1 h at 100°C and subsequently evacuated and sealed. The silica tube was treated in a muffle furnace at 500°C for 100 h followed by slow cooling at a rate of 10°C/h to room temperature. The synthesis products were a mixture of red single crystals of Co_2_V_2_Se_2_O_11_ and a brown-red powder of undetermined composition.

### Refinement

The structure was solved using SHELXS97 (Sheldrick, 2008), and refined by full-matrix least-squares using SHELXL97 (Sheldrick, 2008).

### Crystal data

**Table:**

Co_2_V_2_Se_2_O_11_	*F*(000) = 512
*M**_r_* = 553.66	*D*_x_ = 4.155 Mg m^−^^3^
Monoclinic, *P*2_1_/*m*	Mo *K*α radiation, λ = 0.71073 Å
Hall symbol: -P 2yb	Cell parameters from 1387 reflections
*a* = 4.7913 (2) Å	θ = 3.9–32.2°
*b* = 8.8680 (4) Å	µ = 14.01 mm^−^^1^
*c* = 10.6156 (5) Å	*T* = 292 K
β = 101.115 (5)°	Block, red
*V* = 442.59 (3) Å^3^	0.05 × 0.03 × 0.02 mm
*Z* = 2	

### Data collection

**Table:**

Oxford Diffraction Xcalibur Sapphire3 diffractometer	1534 independent reflections
Radiation source: fine-focus sealed tube	1023 reflections with *I* > 2σ(*I*)
Graphite monochromator	*R*_int_ = 0.051
Detector resolution: 16.5 pixels mm^-1^	θ_max_ = 32.3°, θ_min_ = 3.9°
ω scans	*h* = −7→7
Absorption correction: analytical [*CrysAlis RED* (Oxford Diffraction, 2007), based on expressions derived by Clark & Reid (1995)]	*k* = −11→12
*T*_min_ = 0.659, *T*_max_ = 0.756	*l* = −15→13
4219 measured reflections	

### Refinement

**Table:**

Refinement on *F*^2^	0 restraints
Least-squares matrix: full	Primary atom site location: structure-invariant direct methods
*R*[*F*^2^ > 2σ(*F*^2^)] = 0.032	Secondary atom site location: difference Fourier map
*wR*(*F*^2^) = 0.048	*w* = 1/[σ^2^(*F*_o_^2^) + (0.0114*P*)^2^] where *P* = (*F*_o_^2^ + 2*F*_c_^2^)/3
*S* = 0.76	(Δ/σ)_max_ = 0.001
1534 reflections	Δρ_max_ = 1.28 e Å^−^^3^
85 parameters	Δρ_min_ = −0.98 e Å^−^^3^

### Special details

**Table:**

Geometry. All e.s.d.'s (except the e.s.d. in the dihedral angle between two l.s. planes) are estimated using the full covariance matrix. The cell e.s.d.'s are taken into account individually in the estimation of e.s.d.'s in distances, angles and torsion angles; correlations between e.s.d.'s in cell parameters are only used when they are defined by crystal symmetry. An approximate (isotropic) treatment of cell e.s.d.'s is used for estimating e.s.d.'s involving l.s. planes.
Refinement. Refinement of *F*^2^ against ALL reflections. The weighted *R*-factor *wR* and goodness of fit *S* are based on *F*^2^, conventional *R*-factors *R* are based on *F*, with *F* set to zero for negative *F*^2^. The threshold expression of *F*^2^ > σ(*F*^2^) is used only for calculating *R*-factors(gt) *etc*. and is not relevant to the choice of reflections for refinement. *R*-factors based on *F*^2^ are statistically about twice as large as those based on *F*, and *R*- factors based on ALL data will be even larger.

### Fractional atomic coordinates and isotropic or equivalent isotropic displacement parameters (Å^2^)

**Table:**

	*x*	*y*	*z*	*U*_iso_*/*U*_eq_	
Se1	0.38839 (12)	0.2500	0.45047 (6)	0.00668 (13)	
Se2	0.14374 (12)	0.2500	0.89926 (5)	0.00653 (13)	
Co1	0.95757 (12)	0.42816 (6)	0.63219 (5)	0.00751 (13)	
V1	0.53298 (15)	0.56239 (8)	0.85002 (6)	0.00660 (15)	
O1	0.6860 (8)	0.2500	0.5658 (4)	0.0105 (9)	
O6	0.7568 (6)	0.4597 (3)	0.7887 (3)	0.0118 (6)	
O5	0.3738 (6)	0.4001 (3)	0.9523 (3)	0.0107 (6)	
O2	0.2107 (6)	0.0989 (3)	0.4958 (3)	0.0093 (6)	
O3	0.1701 (8)	0.2500	0.7454 (4)	0.0088 (9)	
O4	0.2448 (6)	0.5786 (3)	0.7407 (3)	0.0132 (7)	
O7	0.6843 (8)	0.7500	0.8501 (4)	0.0077 (9)	

### Atomic displacement parameters (Å^2^)

**Table:**

	*U*^11^	*U*^22^	*U*^33^	*U*^12^	*U*^13^	*U*^23^
Se1	0.0068 (3)	0.0069 (3)	0.0064 (3)	0.000	0.0014 (2)	0.000
Se2	0.0080 (3)	0.0051 (3)	0.0063 (3)	0.000	0.0008 (2)	0.000
Co1	0.0080 (3)	0.0063 (3)	0.0082 (3)	0.0001 (2)	0.0016 (2)	0.0001 (2)
V1	0.0078 (3)	0.0063 (3)	0.0058 (3)	−0.0003 (3)	0.0017 (3)	0.0000 (3)
O1	0.008 (2)	0.005 (2)	0.017 (2)	0.000	−0.0012 (18)	0.000
O6	0.0140 (16)	0.0077 (15)	0.0148 (16)	0.0010 (12)	0.0059 (13)	−0.0014 (12)
O5	0.0153 (16)	0.0113 (15)	0.0045 (14)	−0.0088 (12)	−0.0003 (12)	−0.0024 (11)
O2	0.0106 (15)	0.0103 (16)	0.0071 (15)	−0.0021 (12)	0.0021 (12)	−0.0024 (11)
O3	0.012 (2)	0.006 (2)	0.007 (2)	0.000	−0.0022 (17)	0.000
O4	0.0139 (16)	0.0101 (16)	0.0132 (16)	−0.0020 (12)	−0.0031 (13)	0.0025 (12)
O7	0.009 (2)	0.005 (2)	0.010 (2)	0.000	0.0024 (17)	0.000

### Geometric parameters (Å, º)

**Table:**

Se1—O1	1.691 (4)	Co1—O6	2.093 (3)
Se1—O2	1.706 (3)	Co1—O4^iv^	2.095 (3)
Se1—O2^i^	1.706 (3)	Co1—O3^iv^	2.122 (3)
Se2—O3	1.663 (4)	V1—O4	1.630 (3)
Se2—O5	1.750 (3)	V1—O6	1.635 (3)
Se2—O5^i^	1.750 (3)	V1—O7	1.8147 (17)
Co1—O2^ii^	2.074 (3)	V1—O5	2.038 (3)
Co1—O1	2.081 (3)	V1—O5^v^	2.086 (3)
Co1—O2^iii^	2.090 (3)		
			
O1—Se1—O2	101.12 (13)	O2^ii^—Co1—O3^iv^	91.59 (13)
O1—Se1—O2^i^	101.12 (13)	O1—Co1—O3^iv^	80.04 (12)
O2—Se1—O2^i^	103.52 (19)	O2^iii^—Co1—O3^iv^	172.01 (14)
O3—Se2—O5	98.85 (13)	O6—Co1—O3^iv^	83.90 (13)
O3—Se2—O5^i^	98.85 (13)	O4^iv^—Co1—O3^iv^	88.29 (11)
O5—Se2—O5^i^	98.97 (19)	O4—V1—O6	107.24 (15)
O2^ii^—Co1—O1	95.00 (14)	O4—V1—O7	101.71 (16)
O2^ii^—Co1—O2^iii^	80.44 (12)	O6—V1—O7	102.62 (15)
O1—Co1—O2^iii^	101.07 (12)	O4—V1—O5	95.11 (13)
O2^ii^—Co1—O6	171.78 (11)	O6—V1—O5	99.22 (13)
O1—Co1—O6	90.99 (14)	O7—V1—O5	146.92 (14)
O2^iii^—Co1—O6	103.94 (11)	O4—V1—O5^v^	133.61 (14)
O2^ii^—Co1—O4^iv^	92.69 (12)	O6—V1—O5^v^	117.38 (13)
O1—Co1—O4^iv^	166.17 (13)	O7—V1—O5^v^	81.09 (14)
O2^iii^—Co1—O4^iv^	91.51 (11)	O5—V1—O5^v^	66.81 (12)
O6—Co1—O4^iv^	80.34 (11)		

Symmetry codes: (i) *x*, −*y*+1/2, *z*; (ii) *x*+1, −*y*+1/2, *z*; (iii) −*x*+1, *y*+1/2, −*z*+1; (iv) *x*+1, *y*, *z*; (v) −*x*+1, −*y*+1, −*z*+2.
